# Isolated microorganisms from Iranian grapes and its derivatives

**Published:** 2012-03

**Authors:** S Maulani, SM Hosseini, A Elikaie, SM Mirnurollahi

**Affiliations:** Department of Microbiology, Faculty of Biological Sciences, Shahid Beheshti University, G.C., Evin, Tehran 19839, Iran

**Keywords:** Grapes, Iran, Grape derived products, Bacterium, Fungus

## Abstract

**Background and Objectives:**

The objective of this study was to monitor the microorganisms isolated from grapes and its derivative traditional products produced in Iran.

**Material and Methods:**

Four kinds of grapes cultivated summer of 2010 in vineyard of Takestan and also grape derived products from Shahrod, Hamedan and Takestan were used for this study. The samples were cultured in specific media to isolate the microorganisms that might grow on or pollute the products.

**Results:**

Species of bacteria and fungi isolated from 4 kinds of grapes cultivated in Takestan graveyards and also from 2 kinds of derived traditional products; grape sap and sour grape (abe-ghure locally named), were taken from Takestan, Shahrod and Hamedan cities. Also, bacteria *Bacillus spp*., *Micrococcus spp*., *Clostridium spp*., and fungus of *Penicillium spp*., and *Aspergillus spp*. were isolated.

**Conclusion:**

The isolated bacteria were common microorganisms that grow in soil or in the organic fertilizer and may appear from the environments that samples were collected. These bacteria were not pathogenic to human. The fungus isolated from the grapes may harm humans as they produce toxin. The results suggested that bacterial diversity on grapes and its derived traditional products are expected to be monitored and described in all Iranian graveyards as Iran has been known as one of the world's biggest grape producers.

## INTRODUCTION

Grape is one of the most well-known fruits in the world since centuries ago. In Iran, a grape tree is known as Tak or Mu and based on plant taxonomy; possesses so many different species, which the most important species is *Vitis vinifera*. Its sub species have the best quality product and have been cultivated around the world. The word “Mu” means the unripe grape and its leaves are the source of verjuice and grape syrup. Until 2003, based on its geographic and climate condition, Iran was one of the important regions and the 5^th^ country in the world which produced large volumes of grapes after Italy, USA, Spain, and China (1).

Grape represents a natural reservoir of bacterial species that may influence wine production and storage. Different bacteria may be transferred from grapes into the winery and perturb the vinification process (2). Yeasts, bacteria and filamentous fungi all contribute to the microbial ecology of wine production and the chemical composition of wine, although yeasts have the dominating influence because of their role in conducting the alcoholic fermentation.

Grapevine can be attacked by a number of fungi and fungus-like organisms which affect the berries and cause loss of quality and influence the taste of the wine. Due to attack of the grapevine by pathogens, the infected plant tissue is destroyed and necrotization occurs. When large areas of the canopy are affected by grapevine diseases, the assimilation capacity of the vine is reduced and as a result the berry quality decreases. Aside from leaves, most grapevine pathogens also infect inflorescences, clusters and berries so that the yield can be reduced. Berry infections result in decay of fruit tissue, however, specific effects on berry quality depends on the ripening stage at which the infection occurs (3).

Many studies related to isolation of microorganisms from grapes and wine making process have been carried out in the World, however, no such study has been conducted in Iran so far. The aim of this study was to identify microorganisms; bacteria, fungi and yeast isolated from the grapes grown in different vineyards of Iran and also from 2 kinds of Iranian traditional grape derivative products.

## MATERIAL AND METHODS

### Sample collection

Four kinds of grapes (Shahan, Seedless Green, Seedless red, and Askari) cultivated in vineyard of Takestan, Qazvin province, and Central Iran were used for this study. Takestan is one of the most representative areas of Iranian vineyard, located in about 161 km west of Tehran, capital city of Iran. Samples were taken during summer 2010. Grape syrup samples were taken from 3 cities of Iran; Takestan (central of Iran), Hamedan (west of Iran) and Shahrod (North-East of Iran). Sample of verjuice was taken from Takestan of Qazvin province.

### Grape treatment and culture

20 gr of each sample was added to 90 ml of Nutrient Broth (NB) (Merck 1.05443) and 90 ml of De Man Rogosa and Sharpe Broth (MRS Broth) (Merck 1.10661) medium as enrichment culture. Both medium were supplemented with 100 mg/L of cycloheximide (Tetrachem 601201) to prevent the growth of yeast and fungi. Two kinds of treatment were made, one treatment of samples remained unwashed, and one treatment of samples previously washed with sterile distilled water. After 5 days of incubation at 25°C, rinses with sterile distilled water from grape samples were serially diluted and 1 ml of each dilution inoculated into the surface of the Nutrient Agar (NA) (Merck 1.10416), Plate Count Agar (PCA) (Merck 1.05463) and MRS Agar (Merck 1.10660) by spreading method. After 5–7 days of incubation at 25°C, the colonies were observed and identified using cellular morphology, gram staining method and biochemical tests (4). The isolation and enrichment culture methods were adapted from Bae SS (5).

### Bacterium isolation procedures for grapes’ derivative products A

1 ml of each samples of grape syrup (shire-h angur locally named) and verjuice (abe ghure anggur locally named) were serially diluted followed by inoculation into the surface of the NA and MRS Agar, spreading method. Samples were incubated for 5–7 days at 25°C of incubator under aerobic condition, and then were observed, isolated and identified based on the macroscopic & microscopic findings.

### Isolation of fungi

Fungi isolated from culture were transferred into specific medium for fungi and yeast; Yeast Extract-Glucose-Chloramphenicol Agar (YGCA Merck 1.16000) and Potato Dextrose Agar (PDA Merck 1.10130). Isolated fungi were identified until genus level only (6 and 7). Incubation period were set for 5–7 days.

## RESULTS

From 4 kinds of unprocessed grapes, 3 samples of grape syrup and one sample of verjuice, *Bacillus mycoides, Micrococcus spp., Clostridium spp*., P*enicillium spp*., *Aspergillus spp*., isolated and identified (Table 1).

**Table 1:** Isolated microorganisms from grapes and its traditional derivative products.

Sample/Treatment	Microorganism Type
Grape syrup from Qazvin	Bacille gram (−)
Grape syrup from Shahrod	Bacille gram (−) & *Aspergillus spp*.
Grape syrup from Hamedan	*Clostridium spp. & Aspergillus spp*.
Verjuice from Takestan	Bacille gram (−) & *Aspergillus spp*.
Askari Grape before wash	*Bacillus mycoides*, Bacille gram (−) & *Penicillium spp*.
Seedles Green Grape before wash	Bacille gram (−) & *Micrococcus spp*.
Seedless Red Grape before wash	*Micrococcus spp*., Bacille gram (−) & *Aspergillus spp*.
Shahan Grape before wash	*Bacillus mycoides*, Bacille gram (−) & *Aspergillus spp*.
Askari Grape after wash	*Clostridium spp*., Bacille gram (−), *Penicillium spp., Penicillium spp., & Aspergillus spp*.
Seedless Green Grape after wash	Bacille gram (−)
Seedless Red Grape after wash	Bacille gram (−)
Seedless Red Grape after wash	*Penicillium spp*.
Shahan Grape after wash	Bacille gram (−) & *Penicillium spp*.

The identification of *Bacillus mycoides* was done until second stage of Biochemical tests which results are shown in Table 2.

**Table 2:** Biochemical test of *Bacillus mycoides*.

Name of Test	Bacillus isolated	(1)	(2)	(3)	(4)	(5)	(6)	Matches
Gram reaction	(+)	(+)	D	D	(+)	(+)	(+)	(1) (2) (3) (4) (5) (6)
Motility*	(−)	(−)	(+)	(+)	(+)	(+)	(+)	(1)
Spore position & shape	VX	VX	VX	VX	VX	VTX	TYX	(1) (2) (3) (4) (5)
Growth in 10% NaCl	(+)	D	D	D	(−)	(−)	(+)	(1) (2) (3) (6)
Anaerobic growth	(+)	(+)	(+)	(−)	(+)	(+)	(+)	(1) (2) (4) (5) (6)
Glucose test	(+)	(+)	(+)	(+)	(+)	(+)	(+)	(1) (2) (3) (4) (5) (6)
Galactose test	(+)	D	(−)	D	(+)	D	(−)	(1) (3) (4) (5)
Salicin test	(+)	D	(+)	(+)	(+)	D	D	(1) (2) (3) (4) (5) (6)
Xylose test	(−)	(−)	(−)	D	(+)	D	(−)	(1) (2) (3) (5) (6)
ONPG	(−)	D	(−)	(+)	(+)	D	D	(1) (2) (5) (6)
Urease	(−)	D	D	(−)	D	(−)	(−)	(1) (2) (3) (4) (5) (6)
VP	(+)	(+)	(+)	(+)	D	D	D	(1) (2) (3) (4) (5) (6)
Indole	(−)	(−)	(−)	(−)	(−)	(−)	(−)	(1) (2) (3) (4) (5) (6)
Nitrate	(+)	(+)	(+)	(+)	(+)	D	D	(1) (2) (3) (4) (5) (6)
Starch hydrolysis	(+)	(+)	(+)	(+)	(+)	(+)	(−)	(1) (2) (3) (4) (5)
Oxidase	(+)	D	D	(−)	(−)	(−)	D	(1) (2) (6)
Percentage	100%	100%	87.50%	81.25%	75%	87.50%	75%	

(1) *Bacillus mycoides*, (2) *Bacillus cereus*, (3) *Bacillus subtilis*, (4) *Bacillus licheniformis*, (5) *Bacillus coagulans*, (6) *Bacillus pantothenticus*.

There was no Lactic Acid bacteria (LAB) found in the isolated microorganisms as there was no bacteria grew on MRS Agar, specific medium for LAB isolation.

The fungus species isolated from this study are common species that came from the environments where the samples were taken and the grapes grown very closely to the soil and might be polluted by the organic fertilizer used by the farmer.

## DISCUSSION

In a previous study carried out in Greece, there was no bacterial growth detected on MRS Agar. While in GYC Agar (glucose 5%, yeast extract 1%, CaCO_3_ and agar 2%), relatively high and comparable number were encountered between sound and Botrytis-infected samples of the two grapevine cultivars (2). This study isolated 13 species of bacteria using sequencing and phylogenetic analyses such as *Bacillus subtilis, Staphylococcus plantarum*, and much controversy. Also, *B. mycoides* and the insect pathogen *B. thuringiensis* should be regarded as sub-species of *B. cereus* (2). This is because they differ from *B. cereus* in only a few characteristics which are virtually all plasmid-associated and can be lost, where upon they become indistinguishable from *B. cereus* by the usual characterization tests. In the previous study done in Australia, Sung Sook Bae (5) found the higher populations of *Bacillus thuringiensis* as a biological pesticide and was the most *Gluconobactercerinus* (data not shown).

*Bacillus spp*. is widely distributed in the natural environments. Their unique nature lies in their ability to inhabit a variety of extreme and contaminated environments. Their tolerance to stresses is attributed to their external shield, which is the cell membrane and internal enzymatic system, besides its spore coat which protects it against physical and chemical agents (8) The *Bacillus mycoides* found in this study may be related to which may come from the contaminated environments of vineyards where the grape samples were collected. The *B. cereus, B. anthracis, B. mycoides* and *B. thuringiensis* are all closely related species which have been the subject of much controversy. Also, *B. mycoides* and the insect pathogen *B. thuringiensis* should be regarded as sub-species of *B. cereus* (2). This is because they differ from *B. cereus* in only a few characteristics which are virtually all plasmid-associated and can be lost, where upon they become indistinguishable from *B. cereus* by the usual characterization tests. In the previous study done in Australia, Sung Sook Bae (5) found the higher populations of *Bacillus thuringiensis* as a biological pesticide and was the most prevalent bacterial species on wine grapes throughout cultivation. But LAB and acetic bacteria were rarely detected on undamaged grape. A typical hot and dry condition during cultivation may account for the low populations of bacteria found on wine grapes (5).

In the study done by Heyndrickx (8), several species of *Clostridium* have been isolated from soil; e.g. 16 species in Korean soil from 5 different areas, 54 species in 117 soil samples (on average 7.1 species per sample) from different geographic zones in Costa Rica. This indicates that soil can be a potential reservoir of harmful clostridia species (9). Meanwhile Bae (1) have stated that the potential for acid-tolerant, ethanol tolerant species of *Bacillus* and Clostridium to grow in wines should not be underestimated.

There are several factors to shape the grape micro-biota, including rainfall, temperature, grape variety, berry maturity, sanitary status, and the application of agrichemicals (10). Therefore, microbial diversity in grapes may be quite divergent and associated with the particular vineyard (2).

Previous study on grapes contaminated by moulds and yeasts in USA, found that species such as *Penicillium spp., Aspergillus carbonarius* and yeast (*Rhodotolura*) grew on green seedless grape. Meanwhile *Aspergillus niger*, *Altrenaria* and *Botrytis cinerea* isolated from Red seedless grape. *Penicillium* grew on 10% of green seedless, that many *Penicillium spp*. can also grow at low temperatures and possibly produce mycotoxin on the decayed fruits. *Aspergillus carbonarius* was found in 55 of green seedless whereas *Aspergillus niger* was isolated from 5% of red seedless grapes (11).

In Iran, as previously mentioned, such a study to determine or identify the bacterial micro biota contaminated grapes and its traditional derivative products have never been done before. The grapes used for this study were taken from vineyards where the grape trees laid on the ground so that the grapes collected were covered with dust and/or fertilizer. The weather was dry and hot (about 40°C); meanwhile Takestan vineyards environments have less rainfall especially in summer time. French Mediterranean southern vineyards had a large fungal flora, especially of black aspergilla (*Aspergillus niger*). These fungi were reported to be resistant to high sun exposure and to very hot, frequently achieving temperatures of 40°C during summer season, and dry environments with low rainfall levels characterizing this climate (12).

Limited fungal growth on grapes can be explained by the fact that grapes possess a very hard, smooth skin which protects the vulnerable, nutritional inner tissues from fungal invasion since most fungi causing disease are not capable of breaking the skin barrier. Additionally, grapes are sprayed with fungicides very near the harvest time; such pesticide residues remaining on the fruit during marketing protect against fungal spoilage (11).

In summary, *Bacillus mycoides, Micrococcus spp*., and *Clostridium spp*, have been isolated, and identified using biochemical test. These bacteria are commonly isolated from the soil and may appear from contamination of fertilizer or fungicide used by the farmer or from the environments where the samples were collected. Meanwhile, fungi from the mycotoxin producing genera of *Penicillium* and *Aspergillus* were present and capable of growing in Nutrient Agar and MRS Agar used to isolate the LAB at room temperature. The presence of those fungi on Iranian traditional derivative products of grapes may come from the contamination of non-hygienic environments where the products have been made. These findings are very important to emphasize further research, to find out more about the diversity of bacterial microbiota and fungi that contaminate the grapes all vineyards of Iran.

## References

[1] Zomorrodi S (2005). Storage, Processing and Quality Control of Grapes. Ministry of Jihad-e-Agriculture.

[2] Nisiotou AA, Rantsiou K, Iliopoulos V, Cocolin L, Nychas GE (2011). Bacterial species associated with sound and Botrytis-infected grapes from a Greek vineyard. Int J Food Microbiol.

[3] Kassemeyer HH, Berkelmann-Löhnertz B, König H, Unden G (2009). Fungi of grapes. Biology of microorganisms on Grapes, in Must and in Wine.

[4] Barrow GI, Feltham RKA (2003). Cowan and Steel's manual for the identification of medical bacteria.

[5] Bae SS (2005). Investigation of bacteria associated with Australian Wine Grapes Using Cultural and Molecular Methods.

[6] Ghahry M, Mobasherizadeh S (2010). A Color Atlas of Clinical Laboratory. Urinary sediments, Cytological hematology, Parasitology, Mycology, Bacteriology.

[7] Pitt JI, Hocking AD (1997). Fungi and Food Spoilage.

[8] Heyndrikcx M (2011). the importance of endospore-forming bacteria originating from soil for contamination of industrial food processing. App and Envi Soil Sci.

[9] Gomaa OM, Momtaz OA (2007). 16S rRNA characterization of a Bacillus isolate and its tolerance profile after subsequent subculturing. Arab J Biotech.

[10] Fleet GH (1999). Microorganisms in food ecosystems. Intl J Food Microbiol.

[11] Tournas VH, Katsoudas E (2005). Mould and yeast flora in fresh berries, grapes and citrus fruits. Int J Food Microbiol.

[12] Bejaoui H, Mathieu F, Taillander P, Lebrihi A (2006). Black Aspergilli and ochratoxin A production in French Vineyards. Int J Food Microbiol.

